# A costimulatory molecule-related signature in regard to evaluation of prognosis and immune features for clear cell renal cell carcinoma

**DOI:** 10.1038/s41420-021-00646-2

**Published:** 2021-09-18

**Authors:** Xiaoliang Hua, Shengdong Ge, Jiong Zhang, Haibing Xiao, Sheng Tai, Cheng Yang, Li Zhang, Chaozhao Liang

**Affiliations:** 1 Department of Urology, The First Affiliated Hospital of Anhui Medical University, Hefei, China; 2 Anhui Province Key Laboratory of Genitourinary Diseases, Anhui Medical University, Hefei, China; 3 The Institute of Urology, Anhui Medical University, Hefei, China; 4 Anhui Institute of translational medicine, Hefei, China

**Keywords:** Renal cell carcinoma, Cancer models

## Abstract

Costimulatory molecules have been proven to enhance antitumor immune responses, but their roles in clear cell renal cell carcinoma (ccRCC) remain unexplored. In this study, we aimed to explore the gene expression profiles of costimulatory molecule genes in ccRCC and construct a prognostic signature to improve treatment decision-making and clinical outcomes. We performed the first comprehensive analysis of costimulatory molecules in patients with ccRCC and identified 13 costimulatory molecule genes with prognostic values and diagnostic values. Consensus clustering analysis based on these 13 costimulatory molecular genes showed different distribution patterns and prognostic differences for the two clusters identified. Then, a costimulatory molecule-related signature was constructed based on these 13 costimulatory molecular genes, and validated in an external dataset, showing good performance for predicting a patient’s prognosis. The signature was an independent risk factor for ccRCC patients and was significantly correlated with patients’ clinical factors, which could be used as a complement for clinical factors. In addition, the signature was associated with the tumor immune microenvironment and the response to immunotherapy. Patients identified as high-risk based on our signature exhibited a high mutation frequency, a high level of immune cell infiltration, and an immunosuppressive microenvironment. High-risk patients tended to have high cytolytic activity scores and immunophenoscore of CTLA4 and PD1/PD-L1/PD-L2 blocker than low-risk patients, suggesting these patients may be more suitable for immunotherapy. Therefore, our signature could provide clinicians with prognosis predictions and help guide treatment for ccRCC patients.

## Introduction

Renal cell carcinoma (RCC) is one of the most common malignancies of the urinary system, estimating that 73,750 new cases and 14,830 deaths will occur in the United States in 2020 [1]. Clear cell renal cell carcinoma (ccRCC), the most common histological subtype, is the leading cause of death of RCC patients [2]. A large proportion of patients occurred metastasis at diagnosis owing to lacking characteristic clinical symptoms [3]. Approximately 30% of ccRCC patients developed recurrence and progression despite surgical resection of the primary tumor [4, 5]. Furthermore, ccRCC is chemo- and radio-resistant neoplasia and alternative treatment options have been limited [6]. In recent years, targeted therapies and immunotherapies have further improved the prognosis of ccRCC. However, only a small percentage of ccRCC patients can benefit from these therapies [7, 8]. Therefore, identifications of new biomarkers to predict patients’ survival and response to targeted therapies and immunotherapies are urgently needed.

Immune checkpoint inhibition (ICI) has been added to the armamentarium of metastatic RCC treatment, suggesting that ICI was an effective strategy in the management of RCC [9]. However, the objective response rate was low, and a part of patients experienced drug resistance and disease progression after ICI treatment. Tumor-infiltrating immune cells are thought to be partially relevant to this. Thus, a deeper understanding of the tumor immune microenvironment will help us to improve ccRCC patient outcomes. Previous studies have demonstrated the therapeutic potential of costimulatory molecules in various cancer [10]. Costimulatory molecules play vital roles in the regulation of tumor immunity by affecting the activation and proliferation of T cells [11, 12]. In addition, the most common ICI targets programmed cell death protein 1 (PD-1) and programmed cell death 1 ligand 1 (PD-L1) belong to the B7-CD28 family [13]. These molecules provided potential therapeutic targets for the development of novel ICIs and might play important roles in the regulation of the tumor immune microenvironment [11, 12]. However, the molecular functions of these costimulatory molecules in ccRCC remain unclear.

In the present study, we systematically analyzed the expression patterns and prognostic values of costimulatory molecules in ccRCC. Then, a prognostic signature for ccRCC patients was constructed. The signature was an independent prognostic factor for patients’ prognosis and was characterized by distinct inflammatory profiles and different tumor mutation frequencies. What’s more, we further evaluated the possible response to immunotherapy for different ccRCC patients groups, which was classified according to the costimulatory molecule-based signature.

## Results

### Identification of costimulatory molecule genes with prognostic value in ccRCC

**Fig. 1:**
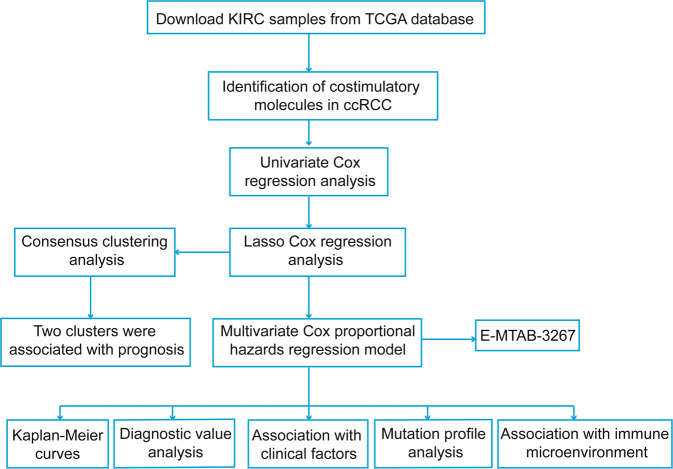
The flowchart of the present study design. The mRNA expression data of tumor and normal tissues were downloaded from TCGA database and ArrayExpress database (E-MTAB-3267). Survival-related costimulatory molecules were obtained and the associations with tumor immune microenvironment were evaluated.

**Fig. 2:**
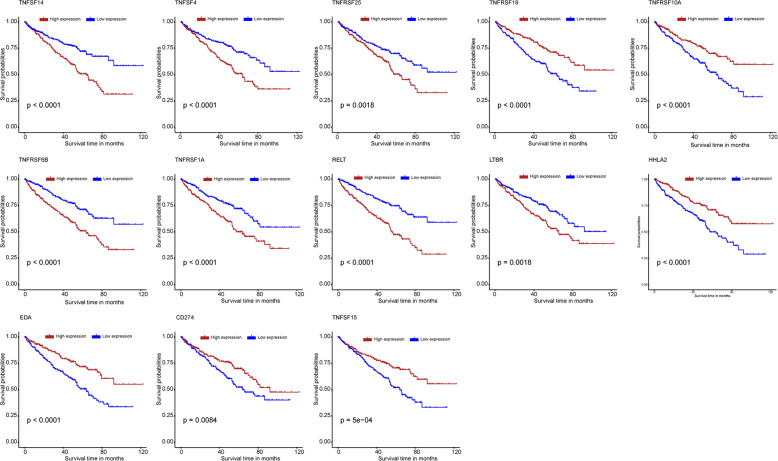
Survival analysis for thirteen costimulatory molecule genes. The Kaplan-Meier curves for the thirteen costimulatory molecule genes in clear cell renal cell carcinoma from The Cancer Genome Atlas dataset, including TNFSF14, TNFSF4, TNFRSF25, TNFRSF6B, TNFRSF1A, RELT, LTBR,TNFRSF19, TNFRSF10A, HHLA2, EDA, CD274, and TNFSF15.

The workflow of this study is demonstrated in Fig. 1. The expression data of 60 costimulatory molecule genes in ccRCC, including 13 B7-CD28 family genes and 47 TNF family genes, were extracted from The Cancer Genome Atlas (TCGA) database. A total of 42 costimulatory molecule genes were significantly associated with the prognosis of ccRCC with *P* < 0.05 (Table S1). These genes were further filtered using the least absolute shrinkage and selection operator (LASSO) analysis, and 13 costimulatory molecule genes were selected (Fig. S1A, B). Kaplan–Meier curves further confirmed the prognostic values of each gene (Fig. 2). High expressions of these genes (TNFSF14, TNFSF4, TNFRSF25, TNFRSF6B, TNFRSF1A, RELT, and LTBR) were associated with a poor prognosis, and low expressions of these genes (TNFRSF19, TNFRSF10A, HHLA2, EDA, CD274, and TNFSF15) were associated with a poor prognosis in ccRCC.

### Cluster classification was associated with the malignancy of ccRCC

**Fig. 3:**
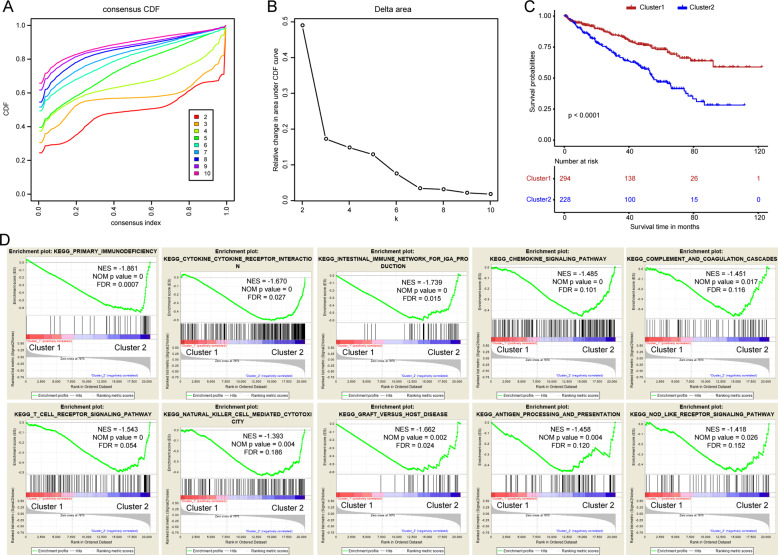
Consensus clustering based on the 13 costimulatory molecule genes. **A** Consensus clustering cumulative distribution function (CDF) for *k* = 2 to *k* = 10. **B** The relative change in area under the CDF curve for *k* = 2 to *k* = 10. **C** Kaplan–Meier curve for two clusters of clear cell renal cell carcinoma. **D** The gene set enrichment analysis showed that several immune-related pathways were significantly enriched in cluster 2.

To explore the overall prognostic value of these genes, we performed a consensus clustering analysis to stratify ccRCC patients. From the results, we found *k* = 3 seemed to be a more stable value from *k* = 2 to 10 (Fig. 3A, B). Then, principal component analysis (PCA) was executed to validate the reliability of the cluster numbers (Fig. S2). We found that these samples had high similarity and gathered together when *k* = 4, and *k* = 5. The number of patients in cluster 2 was small when *k* = 3. Therefore, we divided ccRCC patients into 2 clusters (Fig. S2). Kaplan–Meier curves revealed that patients in cluster 2 showed a worse prognosis than that in cluster 1 (Fig. 3C). What’s more, gene set enrichment analysis (GSEA) showed that several immune-related pathways were significantly enriched in cluster 2 (Fig. 3D).

### Evaluation of the expressions and diagnostic values of costimulatory molecule genes

**Fig. 4:**
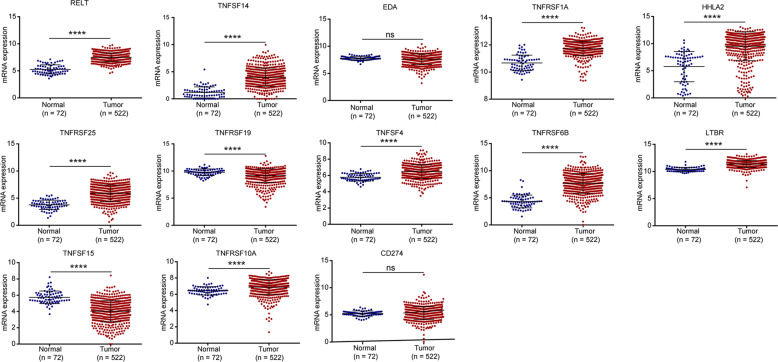
Differential expression of 13 costimulatory molecule genes between normal and tumor tissues. Genes, including RELT, TNFSF14, TNFRSF1A, HHLA2, TNFRSF25, TNFSF4, TNFRSF6B, LTBR, and TNFRSF10A were high-expressed in tumor tissues compared with normal tissues. TNFRSF19 and TNFSF15 were low-expressed in tumor tissues compared with normal tissues. While EDA and CD274 showed no significant difference in tumor tissues compared with normal tissues. ns: *P* > 0.05; *P < 0.05; **P < 0.01; ***P < 0.001; ****P < 0.0001.

The expression levels of 13 costimulatory molecule genes in ccRCC were compared between normal and tumor samples (Fig. 4). Genes, including RELT, TNFSF14, TNFRSF1A, HHLA2, TNFRSF25, TNFSF4, TNFRSF6B, LTBR, and TNFRSF10A had high expression levels, and TNFRSF19 and TNFSF15 had low expression levels in tumor tissues compared with normal tissues. While, genes including EDA, and CD274 showed no significant difference. The diagnostic values of these genes for ccRCC were evaluated (Fig. S3A). Genes, including TNFSF14, TNFRSF25, TNFRSF6B, TNFRSF1A, RELT, LTBR, HHLA2, and TNFSF15 showed excellent diagnostic accuracy with the area under the curve (AUC) > 0.85. Genes, including TNFSF4 and TNFRSF10A, showed well diagnostic accuracy with AUC > 0.70. In addition, the mutation and copy number alteration of these genes were evaluated using the cBioPortal online tool (based on TCGA, Firehose Legacy), 31 samples out of 492 samples were found altered (Fig. S3B). However, the mutation frequencies of all these genes were lower than 2%, suggesting the alterations of mutation and copy number were not the main reason for the expression changes.

### Construction and validation of the prognostic signature based on 13 costimulatory molecule genes

The risk score of a prognostic signature for ccRCC patients was calculated using the expression profiles of 13 costimulatory molecule genes multiplied by the coefficients from multivariate Cox proportional hazards. The detailed formula was showed as follows:

Risk score = (0.24649*RELT) + (0.07762*TNFSF14) + (−0.06576*EDA) + (0.23024*TNFRSF1A) + (−0.14268*HHLA2) + (0.05969*TNFRSF25) + (−0.05832*TNFRSF19) + (0.30355*TNFSF4) + (0.05369*TNFRSF6B) + (0.13797*LTBR) + (−0.02679*TNFSF15) + (−0.06274*TNFRSF10A) + (−0.08476*CD274)

**Fig. 5:**
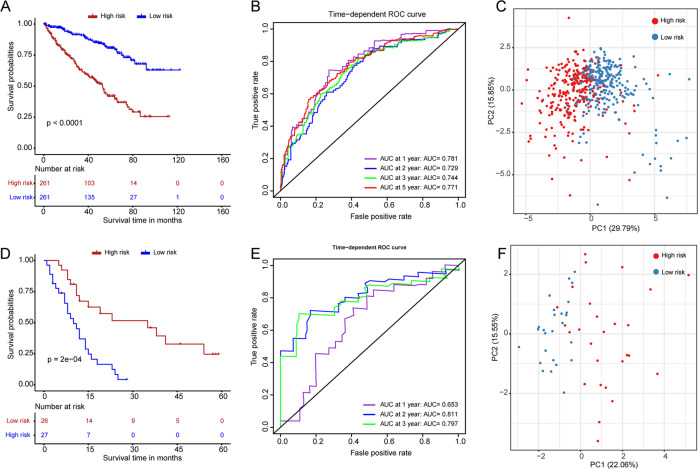
Construction and validation of a costimulatory molecule-based prognostic signature. Kaplan–Meier curves for prognosis evaluation in TCGA dataset (**A**) and E-MTAB-3267 dataset (**D**). Time-dependent receiver operating characteristic curves for the sensitivity and specificity of prognosis evaluation in TCGA dataset (**B**) and E-MTAB-3267 dataset (**E**). Principal component analysis for the evaluation of distribution patterns for high-risk and low-risk patients in TCGA dataset (**C**) and E-MTAB-3267 dataset (**F**).

Patients were divided into high-risk and low-risk groups using the median risk score. Results showed high-risk patients had a poor prognosis compared with low-risk patients (Fig. 5A). A time-dependent receiver operating characteristic (ROC) curve was used to evaluate the performance of the prognostic signature (Fig. 5B). The AUCs of the ROC curves were 0.781 at 1 year, 0.729 at 2 years, 0.744 at 3 years, and 0.771 at 5 years, showing the prognostic signature had moderate sensitivity and specificity. PCA showed different distribution patterns for high-risk and low-risk patients (Fig. 5C). Furthermore, the prognostic signature was further validated in the E-MTAB-3267 dataset. The risk score was calculated using the same formula, and patients were divided into high-risk and low-risk with the median value of the risk score. Kaplan–Meier curve showed high-risk patients had poor prognoses compared with low-risk patients (Fig. 5D). The AUCs of the ROC curves were 0.653 at 1 year, 0.811 at 2 years, and 0.797 at 3 years showing the prognostic signature had moderate sensitivity and specificity (Fig. 5E). PCA analysis showed different distribution patterns for high-risk and low-risk patients in the E-MTAB-3267 dataset (Fig. 5F). These results showed the reliability and stability of the prognostic signature.

### Associations between the prognostic signature and clinicopathological factors of ccRCC

**Fig. 6:**
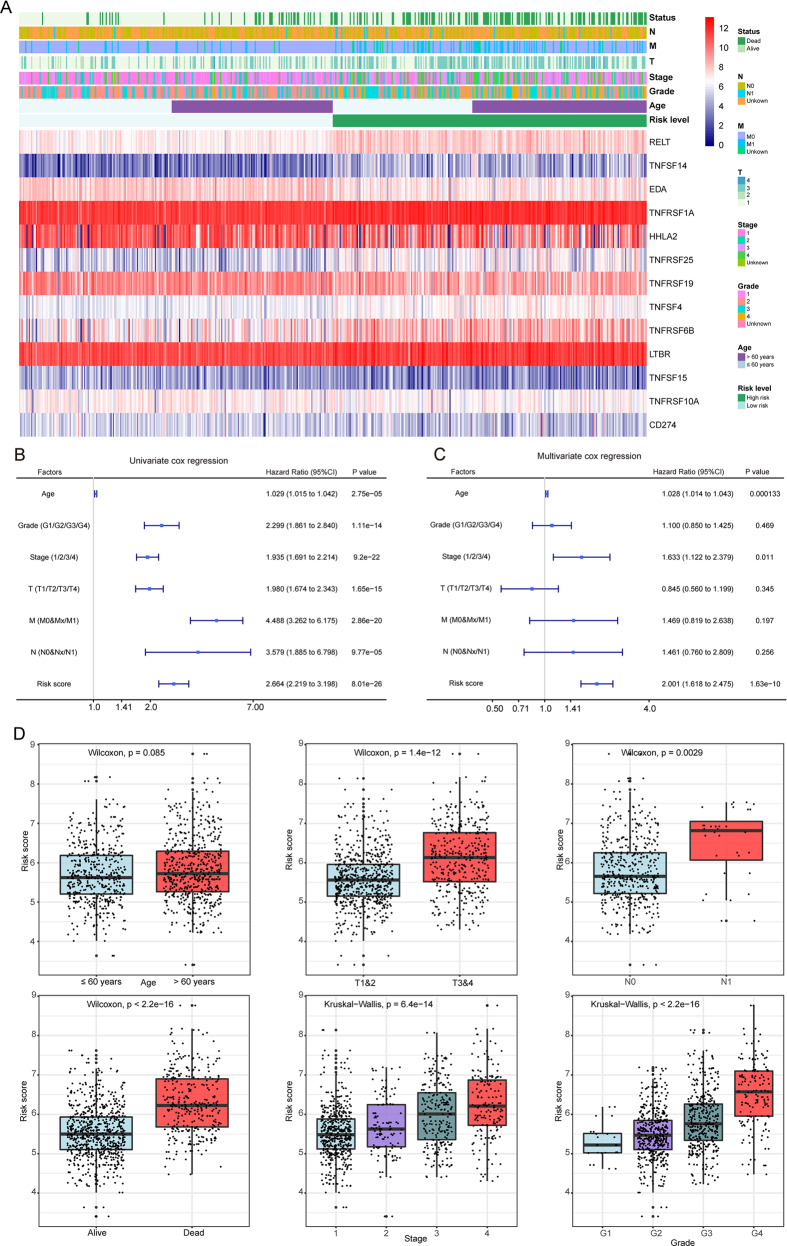
Relationship between the prognostic signature and clinicopathological factors of ccRCC patients. **A** The heat map shows the expressions of the 13 costimulatory molecule genes and clinicopathological factors in the high- and low-risk groups. The univariate (**B**) and multivariate (**C**) Cox regression analyses of prognosis for the prognostic signature and clinicopathological factors. The bar chat showed that the risk score of the prognostic signature in different clinical subgroups. ns: *P* > 0.05, *P < 0.05, **P < 0.01, ***P < 0.001, ****P < 0.0001.

The heat map intuitively showed the expressions of 13 costimulatory molecule genes and the distributions of different clinicopathological factors for ccRCC patients in the high-risk and low-risk groups (Fig. 6A). The associations between the prognostic signature and patients' clinical characteristics were calculated and were shown in Table 1. Univariate Cox regression analysis revealed that age, pathological stage, grade, T stage, N stage, M stage, and risk score were risk factors for patients’ prognosis (Fig. 6B). Multivariate Cox regression analysis showed that the risk score was an independent risk factor for patients’ prognosis (Fig. 6C). Patients were divided into different subgroups according to clinical variables, and we found that ccRCC patients with high stage, high pathological T stage, advanced grade, node metastasis, and dead status tended to have a high-risk score (Fig. 6D). These results demonstrated that our prognostic signature was closely correlated with the clinical factors of ccRCC.

**Table 1:** Association between the risk score of costimulatory molecules signature and patients' clinical characteristics.

Variables	TCGA set (*n* = 522), *n*(%)	Risk score		*P* value
		Low risk (*n* = 261)	High risk (*n* = 261)	
Age (mean ± SD, years)	60.4 ± 12.0	59.7 ± 12.4	61.1 ± 11.6	0.173
*Stage*				<0.001
I	260 (49.8)	165 (63.5)	95 (36.5)	
II	55 (10.5)	30 (54.5)	25 (45.5)	
III	121 (23.2)	46 (38.0)	75 (62.0)	
IV	83 (15.9)	19 (22.9)	64 (77.1)	
Unknown	3 (0.6)	1 (33.3)	2 (66.7)	
*Grade*				<0.001
G1	12 (2.3)	10 (83.3)	2 (16.7)	
G2	225 (43.1)	148 (65.8)	77 (34.2)	
G3	203 (38.9)	87 (42.9)	116 (57.1)	
G4	74 (14.2)	11 (14.9)	63 (85.1)	
Unknown	8 (1.5)	5 (62.5)	3 (37.5)	
*T stage*				<0.001
T1	266 (51.0)	167 (62.8)	99 (37.2)	
T2	67 (12.8)	33 (49.3)	34 (50.7)	
T3	178 (34.1)	60 (33.7)	118 (66.3)	
T4	11 (2.1)	1 (9.1)	10 (90.9)	
*N stage*				0.019
N0/Nx	506 (96.9)	258 (51.0)	248 (49.0)	
N1	16 (3.1)	3 (18.8)	13 (81.2)	
*M stage*				<0.001
M0/Mx	443 (84.9)	243 (54.9)	200 (45.1)	
M1	79 (15.1)	18 (22.8)	61 (77.2)	

### Identification of the prognostic signature-related biological pathways

To explore potential biological pathways for the prognostic signature, genes that strongly correlated with the risk score of the prognostic signature were selected. A total of 670 positively correlated genes and 276 negatively correlated genes were selected, and the results were shown in Fig. S4A. The results of functional-enrichment analysis for Gene Ontology (GO) and Kyoto Encyclopedia of Genes and Genomes (KEGG) pathways were shown in Fig. S4B–E. The most enriched terms for biological process, cellular components, and molecular function were “mitotic nuclear division”, “cytosol” and “protein binding”, respectively (Fig. S4B–D). According to the KEGG analysis, the most significantly enriched term was “Valine, leucine, and isoleucine degradation” (Fig. S4E).

### The associations with the tumor immune microenvironment

**Fig. 7:**
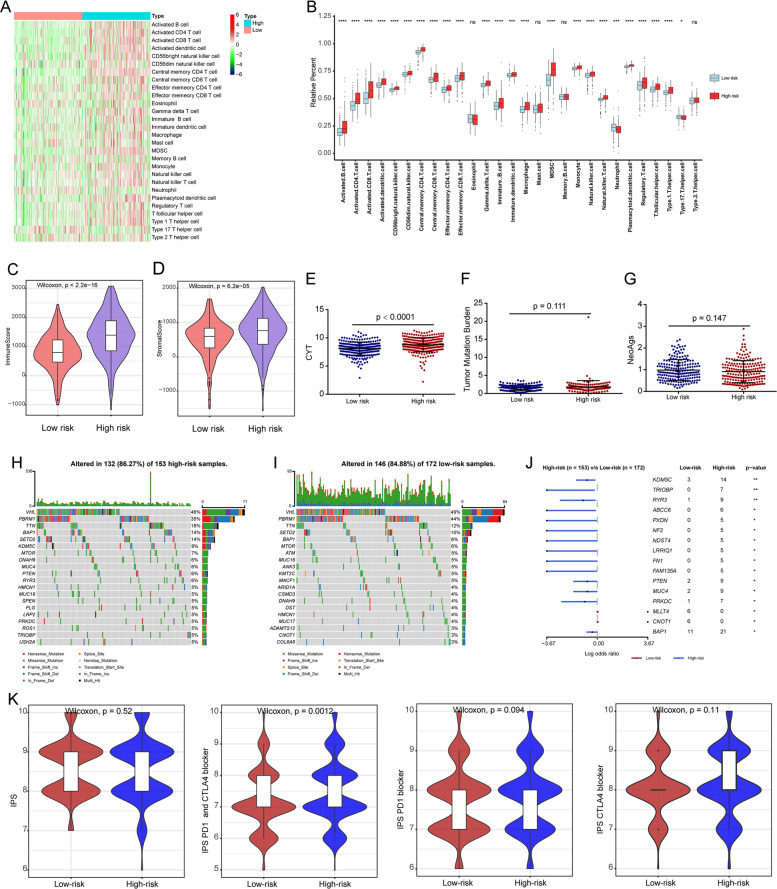
Evaluation of tumor immune microenvironment and genomic alterations. **A** The distributions of 28 immune cells in high-risk and low-risk patients. **B** Box plots showed the detailed differences for 28 immune cells between high-risk and low-risk patients. The differences of the immune score (**C**), stromal score (**D**), cytolytic activity score (**E**), tumor mutation burden (**F**), and neoAgs (**G**) in high-risk and low-risk patients. The mutation profile of the top 20 mutation genes in high-risk patients (**H**) and low-risk patients (**I**). The Forest plot illustrated the differences in mutation frequency of genes in high- and low-risk patients (**J**). Comparison of the IPS, IPS-PD1 blocker, IPS-CTLA4 blocker, and IPS-CTLA4 and PD1 blocker between high-risk and low-risk patients (**K**). *P < 0.05, **P < 0.01, ***P < 0.001, ****P < 0.0001.

The heat map showed significant differences in the immune cell infiltrations between high-risk and low-risk patients (Fig. 7A). The detailed differences for 28 immune cells were shown in the box plots (Fig. 7B). Results showed high-risk patients had a high percentage of various immune cells. Moreover, higher immune scores and stromal scores were found in high-risk patients than that in low-risk patients (Fig. 7C, D).

### Differences in genomic alterations between high-risk and low-risk patients

Results showed that high-risk patients had relatively higher cytolytic activity (CYT) scores (Fig. 7E). However, tumor mutation burden (TMB) and NeoAgs showed no significant difference between high-risk and low-risk patients (Fig. 7F–G). What’s more, the top 20 mutated genes for high-risk (Fig. 7H) and low-risk (Fig. 7I) patients were compared. The mutation frequencies of genes, including KDM5C, TRIOBP, RYR3, ABCC6, PXDN, NF2, NDST4, LRRIQ1, FN1, FAM135A, PTEN, MUC4, PRKDC, and BAP1 were higher, and the mutation frequencies of MLLT4 and CNOT1 genes were lower in a high-risk group (Fig. 7J). The immunophenoscore (IPS) was used to evaluate the response to ICI therapy [14]. We found IPS-CTLA4 and PD1/PD-L1/PD-L2 blockers were slightly higher in the high-risk group compared with the low-risk group. While, the IPS, IPS-PD1/PD-L1/PD-L2 blocker, IPS-CTLA4 blocker showed no difference (Fig. 7K).

## Discussion

CcRCC has shown durable responses to ICI therapies, and nivolumab has been approved as a second-line treatment for metastatic RCC [15]. However, a large part of ccRCC patients does not respond to ICI therapies. Thus, identification of biomarkers for predicting response to ICI therapies and selection of the most sensitive patients are key to increase response rates. Costimulatory molecules have been revealed to play an important role in the progression of various tumors [16–18]. The roles of costimulatory molecules in ccRCC remain to be explored. In the present study, we systematically evaluated the roles of costimulatory molecules in ccRCC and selected 13 genes with prognostic values to further study. Moreover, we constructed and validated a new prognostic signature for ccRCC patients. To our knowledge, the present study provides the first prognostic signature of costimulatory molecules in patients with ccRCC. We found that the prognostic signature was an independent risk factor for ccRCC patients and was significantly correlated with patients’ clinical factors. Additionally, we found that our prognostic signature was associated with the tumor immune microenvironment and the response to immunotherapy, which might provide valuable clues for predicting patients’ prognosis and selections of patients for immunotherapy.

The costimulatory molecules play an important role in the regulation of tumor immunity [19, 20]. Monoclonal antibodies targeted PD-1/PD-L1 (B7-H1) or B7-2/CTLA-4 pathways have shown promise to induce durable tumor regressions in various tumors [21, 22]. All these therapeutic targets belong to costimulatory molecules. To explore the expression levels and prognostic values of costimulatory molecules in ccRCC, we acquired 13 members of the B7-CD28 family and 47 members of the TNF family for ccRCC patients [23, 24]. Thirteen costimulatory molecular genes (TNFSF14, TNFSF4, TNFRSF25, TNFRSF6B, TNFRSF1A, RELT, LTBR, TNFRSF19, TNFRSF10A, HHLA2, EDA, CD274, and TNFSF15) with prognostic values were selected. TNFRSF6B was overexpressed in ccRCC and could promote adhesion, migration, and invasiveness of tumor cells of ccRCC [25]. TNFRSF1A was upregulated in ccRCC patients with tyrosine kinase inhibitor resistance and was an independent predictive factor for unfavorable response to tyrosine kinase inhibitor and shorter survivals [26]. HHLA2 was highly expressed in ccRCC tissues, which could function as a T-cell co-inhibitory factor to play an immunosuppressive effect, promoting tumor migration and invasion [27, 28]. CD274, namely PD-L1, is an effective therapeutic target for ccRCC [29]. The expression of TNFSF15 in ccRCC was markedly decreased and was more likely to be a tumor-suppressive factor [30]. The functions of other costimulatory molecules in ccRCC remain unclear. However, the roles of these genes have been reported in other tumors. TNFSF14, also known as LIGHT, has been used to treat multiple tumors in combination with other immunotherapy modalities [31]. TNFSF4, also known as OX40L, is a co-stimulatory checkpoint protein that could enhance the anti-neoplastic activity of T cells [32]. TNFRSF25, also known as DR3, plays essential roles in protective inflammation, autoimmune diseases, and tumor immunotherapy [33]. RELT is significantly upregulated in glioma and is associated with a poor prognosis [34]. LTBR functions as a potential anti-tumor role by triggering apoptosis of tumor cells or by eliciting anti-tumor immune response [35]. TNFRSF19, also known as TROY, was inversely correlated with patient survival and could stimulate glioblastoma cell migration and invasion [36]. TNFRSF10A, also known as DR4, has been reported to be involved in the pathogenesis of various tumors [37]. These costimulatory molecular genes were new and were needed to be investigated in ccRCC.

With the development of immunotherapy, identification of biomarkers and selection of the most sensitive patients are urgently needed to increase the response rates of immunotherapy. In the present study, 13 costimulatory molecular genes were selected, and consensus clustering analysis was performed to explore the overall prognostic values. Kaplan–Meier curves showed a worse prognosis of these patients in cluster 2. In addition, several immune-related pathways were enriched in cluster 2, suggesting that these selected genes were highly associated with the tumor immune microenvironment. The worse prognosis in patients of cluster 2 might be owing to the deficiency of immune system or immune defense restricted. Moreover, a risk signature based on costimulatory molecular genes may provide new insights for the clinical practice of ccRCC patients. The risk signatures of costimulatory molecular genes have been constructed in colorectal cancer [38], and lung adenocarcinoma [39]. All these prognostic signatures were reliable and showed good performance. As we know, we were the first to construct the risk signature based on costimulatory molecular genes for ccRCC patients. The performance of our prognostic signature was validated in TCGA and E-MTAB-3267 datasets, and all showed good performance. We further found that the prognostic signature is closely correlated with clinical factors, which could be applied as a supplement for guiding treatment. We also selected genes highly correlated with the risk score of our prognostic signature, and functional enrichment analysis for these genes showed T cell homeostasis and NF-κB signaling were enriched.

To further explore the associations between our signature and tumor immune microenvironment, the immune cell infiltration and tumor mutation profiles were compared between high-risk and low-risk patients. Results showed that high-risk patients had significantly greater infiltration of immune cells. In addition, the infiltration of various immunosuppressive cells, including gamma delta T cells, immature dendritic cells, macrophages, monocyte, MDSCs, plasmacytoid dendritic cells, regulatory T cells (Treg), and T follicular helper cells were also greater in high-risk patients, suggesting the presence of an immunosuppressive microenvironment in high-risk patients. The immunosuppressive microenvironment is an important mechanism for tumor cells to escape immune attacks and promote disease progression. MDSCs play vital roles in suppressing the immune responses of T and NK cells and stimulating Treg, propelling tumor immune escape and tumor progression. MDSC can contribute to patient resistance to ICI [40], and can be used to predict the response to sunitinib therapy or cytokine therapy in ccRCC [41, 42]. Understanding the immune microenvironment of each ccRCC patient can help us identify patients who are more likely to benefit from immunotherapy, and combine novel treatment strategies to improve treatment response rates. TMB and the neoantigen load were tumor-intrinsic factors for tumor immunogenicity and could be used as biomarkers for evaluating the response to immunotherapy [43, 44]. The IPS was a superior predictor of response to CTLA-4 and anti-PD-1 antibodies and was validated in two independent cohorts [14]. In the present study, we found high-risk patients tended to have high CYT scores and IPS-CTLA4 and PD1/PDL1/PD-L2 blockers than low-risk patients. The TMB was tended to be higher in the high-risk group compared with the low-risk group despite the *P* value larger than 0.05. These results suggested high-risk patients tended to have a “hot” immune microenvironment and high mutational burden, which might increase immunogenicity, leading to a relatively higher response rate to immunotherapy. However, the clinical study in the real world was needed to confirm the above-speculated results.

Inevitably, there are several limitations in this study. The present study mainly derived from public databases and was retrospective. The amount of available datasets with prognostic information for ccRCC patients is limited so that the clinical parameters analyzed in the present study are not comprehensive. CcRCC patients with prognostic information in the real world are needed to determine the values of the prognostic signature. Secondly, genes enrolled in the present study were restricted to the costimulatory molecules and the immune tumor microenvironment has high spatial heterogeneity. Thus, the power of the prognostic signature was limited. Moreover, the expression data of costimulatory molecular genes in ccRCC patients with immunotherapy was not available. Therefore, the risk signature for evaluating the response to immunotherapy was indirect. Future prospective studies for ccRCC patients with immunotherapy were needed to confirm the clinical application value of our signature.

In conclusion, we performed the first comprehensive analysis of costimulatory molecules in ccRCC patients and identified 13 genes with prognostic and diagnostic values. We constructed and validated a new prognostic signature for ccRCC patients based on costimulatory molecules, and explored its potential molecular mechanisms. Our prognostic signature could stratify patients into two subgroups with different prognoses and showed high associations with the clinical features. Moreover, patients identified as high risk based on our prognostic signature exhibited a high mutation frequency, a high level of immune cell infiltration, and an immunosuppressive microenvironment. Thus, we believed that our signature could provide clinicians with prognosis predictions and treatment guidance for ccRCC patients.

## Materials and methods

### Data collection and preprocessing

The RNA-sequencing data and the corresponding clinical dataset in the TCGA database for ccRCC patients were obtained from UCSC Xena (https://xenabrowser.net/). In addition, a total of 53 ccRCC patients in the E-MTAB-3267 dataset from the ArrayExpress database (https://www.ebi.ac.uk/arrayexpress/) were used to validate our results. The detailed information for data preprocessing is provided in the Supplementary materials and methods.

### Identification of costimulatory molecules with prognostic significance in ccRCC

Univariate Cox regression analysis, Kaplan–Meier curves, and LASSO analysis were conducted to select genes with prognostic values. The detailed information is provided in the Supplementary materials and methods.

### Consensus clustering of survival-related costimulatory molecule genes

To further explore the functions and prognostic values of the costimulatory molecules in ccRCC, consensus clustering was performed with the “ConsensusClusterPlus” R package [45]. The detailed information is provided in the Supplementary materials and methods.

### Construction and validation of a costimulatory molecule-related prognostic signature

The costimulatory molecule-related prognostic signature was constructed, and the detailed formula was shown as follows: Risk score = *β*_1_*Exp_1_ + *β*_2_*Exp_2_ + *β*_i_*Exp_*i*_. *β* and Exp represent the coefficients from the multivariate Cox proportional hazards regression analysis and the expression levels of selected genes, respectively. The detailed information is provided in the Supplementary materials and methods.

### Functional and pathway enrichment analysis

To explore signature-related biological pathways, genes that were strongly correlated with the risk score (correlation coefficient |*R*| > 0.5) were obtained. A total of 670 positively correlated genes and 276 negatively correlated genes were selected. The Database for Annotation, Visualization, and Integrated Discovery (https://david.ncifcrc.gov/) was used to perform GO and KEGG pathway enrichment analysis for these genes [46]. *P* < 0.05 was regarded as the cutoff value.

### Estimation of the immune microenvironment composition

Single-sample gene set enrichment analysis (ssGSEA) was performed to quantify the 28 types of immune cells [14]. The immune and stromal scores for the total TCGA cohorts, reflecting the infiltration levels of non-tumor cells, were calculated using the “ESTIMATE” package [47]. Differences in the immune microenvironment composition were compared for the low-risk and high-risk groups. The detailed information is provided in the Supplementary materials and methods.

### Comparison of significantly mutated genes and response to ICIs

TMB, the CYT score, the somatic mutation status data, and IPS for each ccRCC patient were collected and compared between high-risk and low-risk patients. The detailed information is provided in the Supplementary materials and methods.

### Statistical analyses

We performed a *t*-test or Wilcoxon test for comparisons of two variables. Kaplan–Meier curves and log-rank tests were used to evaluate survival differences. Univariate and multivariate Cox regression analyses were performed to evaluate the prognostic values of costimulatory molecule genes. Pearson’s chi-square test was used to evaluate differences in the distribution of clinical factors for ccRCC patients. All these procedures involved in the present study were conducted on R software. *P* < 0.05 was considered to be statistically significant.

## Supplementary information

Supplementary Materials and Methods

Supplementary Figure and Table legends

Figure S1

Figure S2

Figure S3

Figure S4

Table S1

## Data Availability

The data could be download at (https://portal.gdc.cancer.gov/, https://xenabrowser.net/ and https://www.ebi.ac.uk/arrayexpress/; E-MTAB-3267) and the code used during the current study are available from the corresponding author on reasonable request.
