# Supplementary material for: A costimulatory molecule-related signature in regard to evaluation of prognosis and immune features for clear cell renal cell carcinoma
Source: Cell Death Discov. 2021 Sep 18;7:252. doi: 10.1038/s41420-021-00646-2 (PMC8449780; doi:10.1038/s41420-021-00646-2)
Supplement: Supplementary file 1 — Supplementary Materials and Methods [file 41420_2021_646_MOESM1_ESM.docx]

**Supplementary materials and methods**

**Data collection and preprocessing**

For TCGA dataset of ccRCC patients, the expression data was normalized using the “RNA-Seq by Expectation-Maximization” R package, and was performed log2(x+1) transformed. The samples were filtered based on following criterion: (1) Duplicate samples are removed. (2) Patients with follow-up time less than one week were excluded. At last, 72 normal samples and 522 ccRCC samples were included in present study. For the E-MTAB-3267 microarray data, robust multi-array averaging method was used for background correction and these probes were annotated using corresponding Affymetrix annotation files. All genes were uniformly normalized for these datasets. For repeated genes, their expression levels were calculated as the mean expressions.

**Identification of costimulatory molecules with prognostic significance in ccRCC**

All costimulatory molecule genes were firstly mapped to TCGA dataset, and high- and low-expressed groups of each costimulatory molecule gene were divided based on median values of the expressions. Univariate Cox regression analysis was performed to select the survival-associated costimulatory molecule genes. *P* < 0.05 was set as cutoff value. Kaplan-Meier curves and log-rank tests for prognostic evaluation were performed using the “survminer” R package. To minimize overfitting, LASSO analysis was conducted with “glmnet” package to select the most valuable genes for predicting the survival of ccRCC patients[^1^](#_ENREF_1). The 10-fold cross-validation was used to determine the optimal value of the penalty parameter.

**Consensus clustering of survival-related costimulatory molecule genes**

The algorithm of consensus clustering is provided in “ConsensusClusterPlus” R package[^2^](#_ENREF_2). The algorithm begins by subsampling a proportion of items from a data matrix, and then partitions into up to *k* groups by a user-specified clustering algorithm. This process is repeated for a user-specified number of repetitions. Pairwise consensus values, defined as the proportion of clustering runs in which two items are grouped together, are calculated and stored in a consensus matrix for each *k*. Then, a final agglomerative hierarchical consensus clustering using distance of 1-consensus values is completed and pruned to *k* groups for each *k.* This algorithm is defined as “consensus clusters” which is used to establish consensus values and to assess the stability of the identified clusters. Graphical output results includes heat maps of the consensus matrices, consensus cumulative distribution function (CDF) plots, and delta area plots. The heat maps display the clustering results, where items nearly always either cluster together giving a high consensus (dark blue color) or do not cluster together giving a low consensus (white). The CDF plots and delta area plots allow us to determine an approximate number of clusters. Numbers of clusters were determined based on the following criteria: relatively high consistency within the cluster, relatively low coefficient of variation, and no appreciable increase in the area under the CDF curve. The present study used the following parameters: 1000 repeats, *k* = 10, and agglomerative hierarchical clustering with ward criterion (Ward.D2) inner and complete outer linkage. Genes used for consensus clustering analyses are the thirteen selected costimulatory molecule genes. The cluster numbers were further confirmed using PCA analysis with the “ggplot2” package. Then, Kaplan-Meier curves were plotted to confirm the prognostic value of the cluster classification. GSEA was performed to reveal the potential functional mechanisms using the c2.cp.kegg.v7.2.symbols.gmt file. False discovery rate (FDR) < 0.25 and normalized *P* value < 0.05 were set as the threshold values.

**Construction and validation of a costimulatory molecule-related prognostic signature**

Multivariate Cox proportional hazards regression analysis was performed to obtain the coefficients for these survival-related costimulatory molecule genes. The costimulatory molecule-related prognostic signature was constructed based on the coefficients of multivariate Cox regression analysis weighted with the expression of these selected genes[^3^](#_ENREF_3)^,^[^4^](#_ENREF_4). The detailed formula was showed as follow: *Risk score = β_1_ * Exp_1_ + β_2_ * Exp_2_ + β_i_ * Exp_i_*. *β* and *Exp* represent the coefficients from the multivariate Cox proportional hazards regression analysis and the expression levels of selected genes, respectively. To evaluate the sensitivity and specificity of the prognostic signature, time-dependent ROC analysis was performed, and the AUCs were calculated using the “survival ROC” R package[^5^](#_ENREF_5). According to the median risk score value, patients were then classified into high- and low-risk subgroups. Kaplan-Meier analysis and log-rank test for prognostic evaluation were performed using the “survminer” R package.

**Estimation of the immune microenvironment composition**

To quantify the relative proportion of immune cells in each risk group, a set of metagenes, including non-overlapping sets of genes that are representative of twenty-eight specific immune cell subpopulations, was obtained[^6^](#_ENREF_6). Single-sample gene set enrichment analysis (ssGSEA) was performed to quantify the twenty-eight types of immune cells based on the set of metagenes. Then, we compared the immune cell phenotypes of the low- and high-risk groups. Stromal and immune cells are the two main non-tumor components in the tumor microenvironment, which have been proposed to be valuable in the treatment and prognostic assessment of tumors. To investigate the tumor microenvironment of different risk groups, immune and stromal scores for the total TCGA cohorts reflecting the infiltration levels of non-tumor cells were calculated using the ESTIMATE package[^7^](#_ENREF_7). Differences in the immune and stromal scores of ccRCC were compared for the low- and high-risk groups.

**Comparison of significantly mutated genes and response to ICIs**

TMB is defined as the total amount of coding errors of somatic genes, base substitutions, insertions or deletions detected per million bases[^8^](#_ENREF_8). In the present study, tumor mutation burden and neo-antigen data for TCGA dataset were acquired from the research of Michael S Rooney[^9^](#_ENREF_9). The CYT score, reflecting the intratumoral immune cytolytic T-cell activity, was calculated as the geometric mean of the GZMA and PRF1 expression levels[^9^](#_ENREF_9). The somatic mutation status data of KIRC samples (workflow type: VarScan2 Variant Aggregation and Masking) were downloaded from the TCGA data portal (https://portal.gdc.cancer.gov/repository) in March 2021. Mutation data were filtered using the “maftools” R package and compared between high-risk and low-risk patients. In this study, the IPS for each ccRCC patients were collected from The Cancer Immunome Atlas (TCIA) (<https://tcia.at/home>), and compared between high-risk and low-risk patients.

**References**

1. Tibshirani, R. The lasso method for variable selection in the Cox model. *Stat. Med.* **16**, 385-395 (1997).

2. Wu J., Cui Y., Sun X., Cao G., Li B., Ikeda D. M. et al. Unsupervised Clustering of Quantitative Image Phenotypes Reveals Breast Cancer Subtypes with Distinct Prognoses and Molecular Pathways. *Clin. Cancer. Res.* **23**, 3334-3342 (2017).

3. Lossos I. S., Czerwinski D. K., Alizadeh A. A., Wechser M. A., Tibshirani R., Botstein D. et al. Prediction of survival in diffuse large-B-cell lymphoma based on the expression of six genes. *N. Engl. J. Med.* **350**, 1828-1837 (2004).

4. Chen H. Y., Yu S. L., Chen C. H., Chang G. C., Chen C. Y., Yuan A. et al. A five-gene signature and clinical outcome in non-small-cell lung cancer. *N. Engl. J. Med.* **356**, 11-20 (2007).

5. Heagerty, P. J., Lumley, T., & Pepe, M. S. Time-dependent ROC curves for censored survival data and a diagnostic marker. *Biometrics* **56**, 337-344 (2000).

6. Charoentong P., Finotello F., Angelova M., Mayer C., Efremova M., Rieder D. et al. Pan-cancer Immunogenomic Analyses Reveal Genotype-Immunophenotype Relationships and Predictors of Response to Checkpoint Blockade. *Cell. Rep.* **18**, 248-262 (2017).

7. Yoshihara K., Shahmoradgoli M., Martinez E., Vegesna R., Kim H., Torres-Garcia W. et al. Inferring tumour purity and stromal and immune cell admixture from expression data. *Nat. Commun.* **4**, 2612 (2013).

8. Chalmers Z. R., Connelly C. F., Fabrizio D., Gay L., Ali S. M., Ennis R. et al. Analysis of 100,000 human cancer genomes reveals the landscape of tumor mutational burden. *Genome. Med.* **9**, 34 (2017).

9. Rooney M. S., Shukla S. A., Wu C. J., Getz G., & Hacohen N. Molecular and genetic properties of tumors associated with local immune cytolytic activity. *Cell* **160**, 48-61 (2015).
