# Supplementary material for: A costimulatory molecule-related signature in regard to evaluation of prognosis and immune features for clear cell renal cell carcinoma
Source: Cell Death Discov. 2021 Sep 18;7:252. doi: 10.1038/s41420-021-00646-2 (PMC8449780; doi:10.1038/s41420-021-00646-2)
Supplement: Supplementary file 2 — Supplementary Figure and Table legends [file 41420_2021_646_MOESM2_ESM.docx]

**Supplementary Figure legends**

**Figure S1.** The least absolute shrinkage and selection operator (LASSO) Cox regression analysis based on thirteen costimulatory molecule genes. Plot of the coefficients of LASSO Cox regression analysis (A). Plot of partial likelihood deviance for thirteen costimulatory molecule genes in TCGA dataset. (B)

**Figure S2.** Identification of cluster numbers using consensus clustering. Consensus clustering matrix for *k* = 2, *k* = 3, *k* = 4, and *k* = 5. Principal component analysis for evaluating the distributions of different cluster numbers.

**Figure S3.** The diagnostic values and the mutation and copy number alteration of thirteen costimulatory molecule genes for ccRCC. (A) The diagnostic values of thirteen costimulatory molecule genes to distinguish tumor from normal samples. (B) The mutation and copy number alteration of thirteen costimulatory molecule genes for ccRCC.

**Figure S4.** The costimulatory molecule-based signature-related biological pathways. (A) The most related genes of costimulatory molecule-based signature in clear cell renal cell carcinoma (Pearson |R| > 0.5). The results of functional enrichment analysis for related genes of costimulatory molecule-based signature, including biological process (B), cell component (C), molecular function (D), and KEGG term (E).

**Supplementary Table legends**

**Table S1.** Univariate Cox regression analysis of overall survival for costimulatory molecule genes.
