# Supplementary figures and images for: A costimulatory molecule-related signature in regard to evaluation of prognosis and immune features for clear cell renal cell carcinoma
Source: Cell Death Discov. 2021 Sep 18;7:252. doi: 10.1038/s41420-021-00646-2 (PMC8449780; doi:10.1038/s41420-021-00646-2)

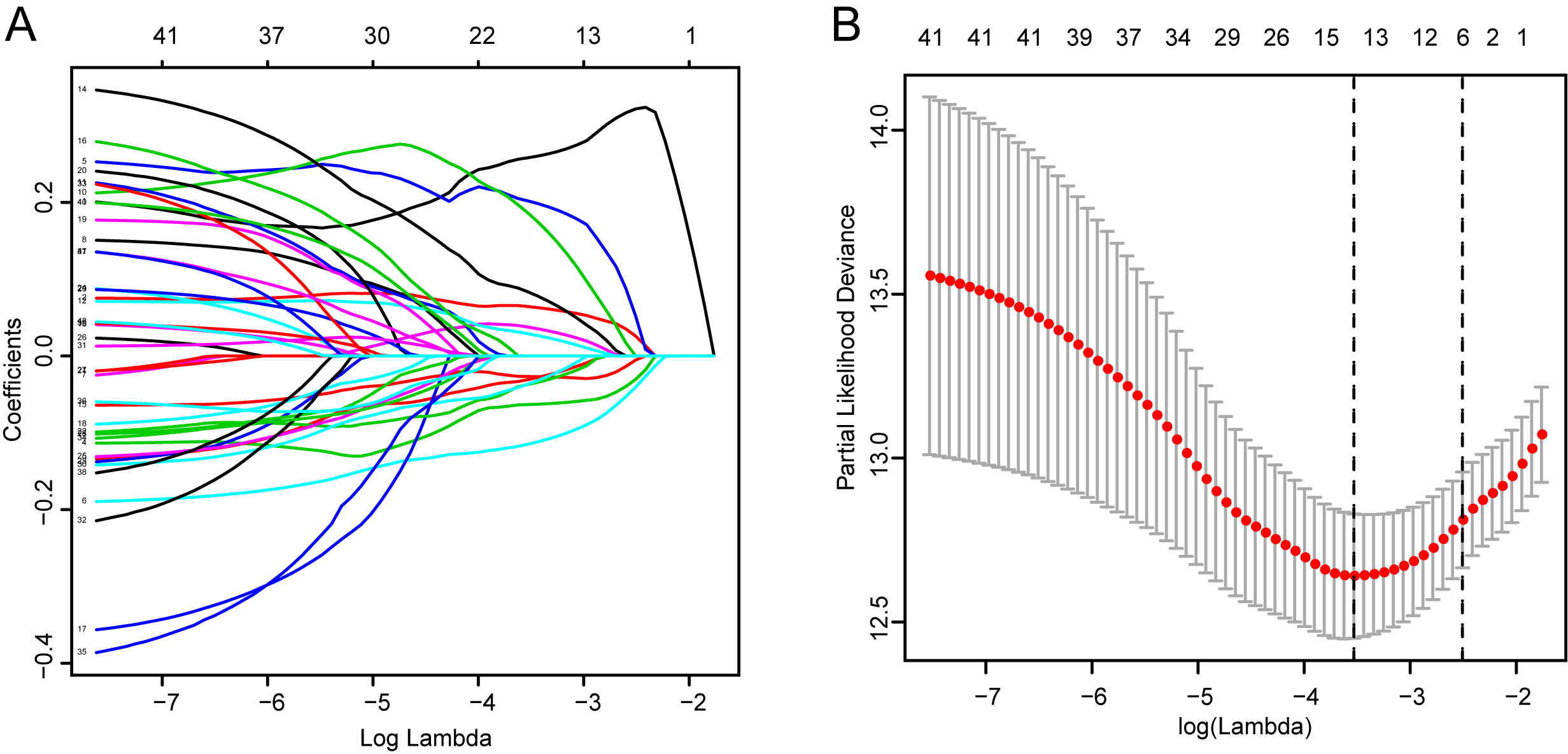

Supplement: Supplementary file 3 — Figure S1 [file 41420_2021_646_MOESM3_ESM.tif]

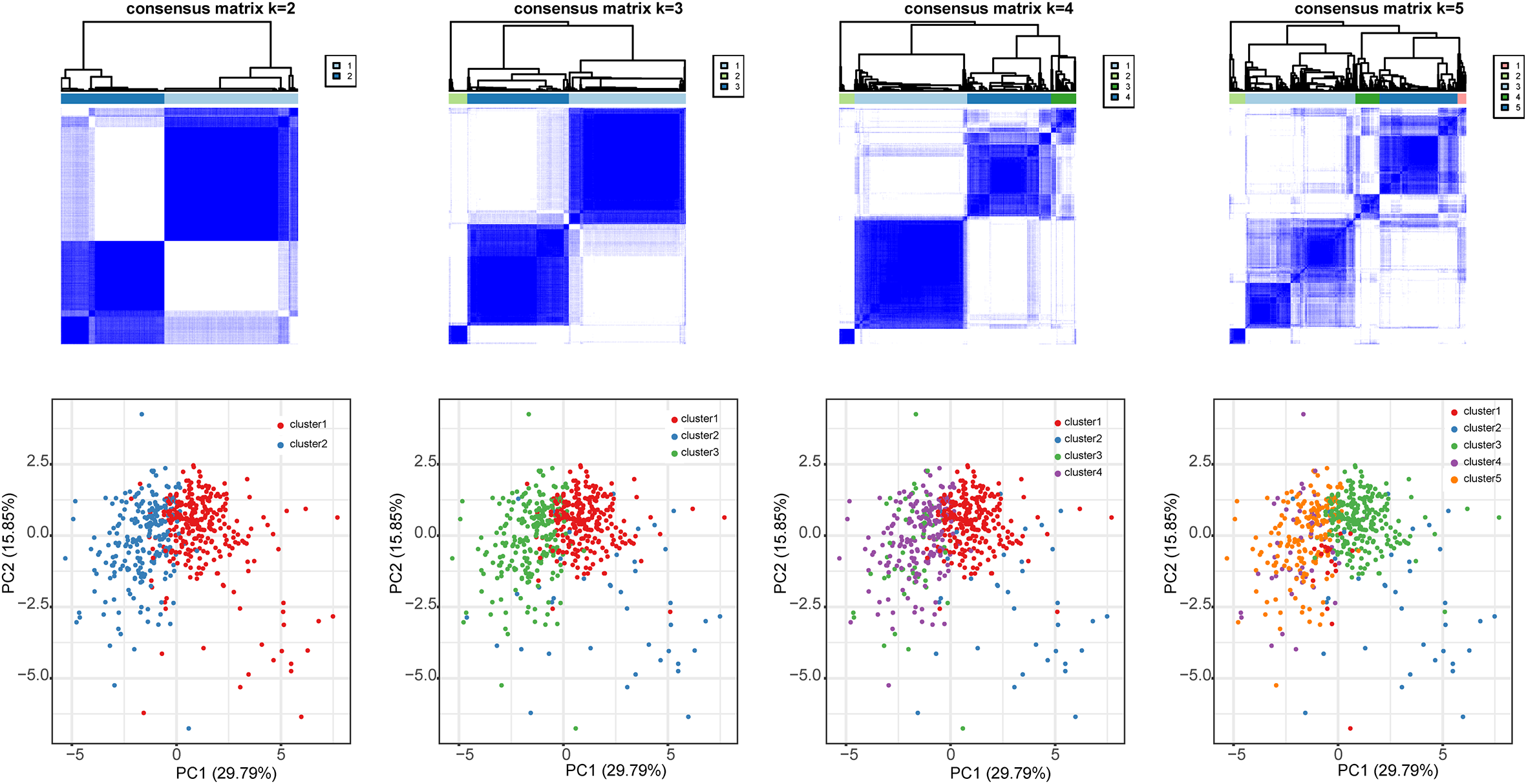

Supplement: Supplementary file 4 — Figure S2 [file 41420_2021_646_MOESM4_ESM.tif]

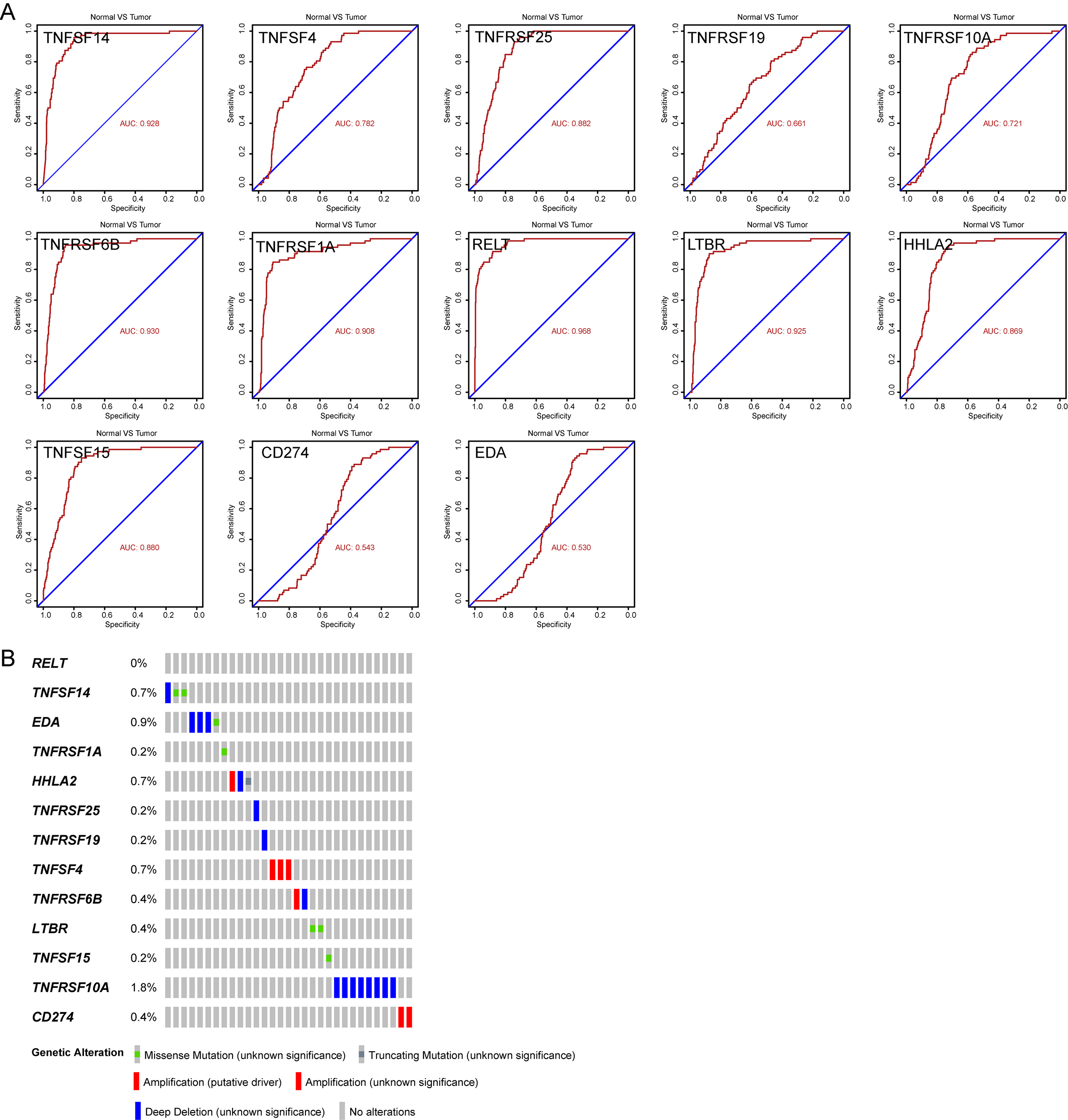

Supplement: Supplementary file 5 — Figure S3 [file 41420_2021_646_MOESM5_ESM.tif]

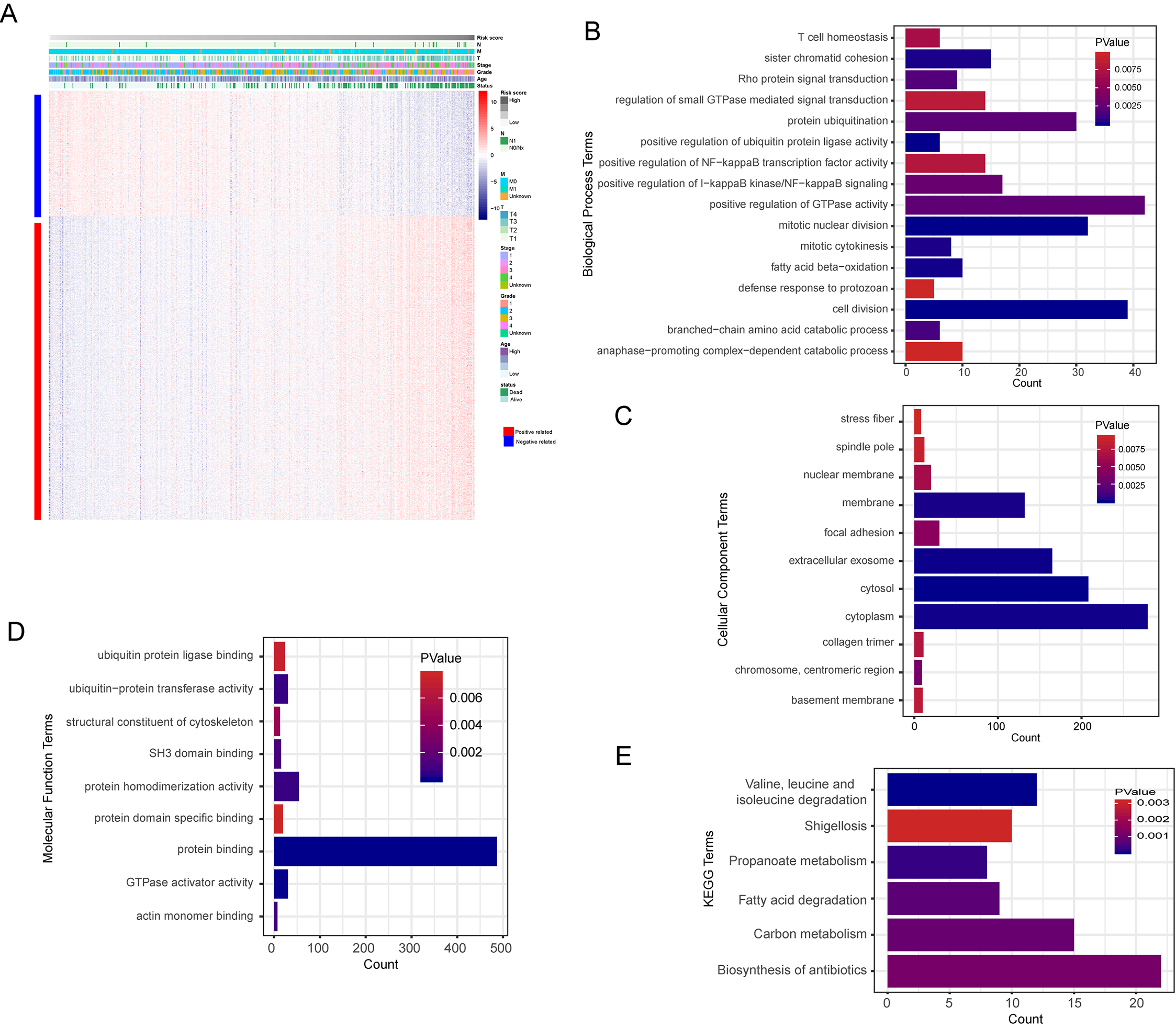

Supplement: Supplementary file 6 — Figure S4 [file 41420_2021_646_MOESM6_ESM.tif]
